# Whole-gland high-intensity focused ultrasound ablation and transurethral resection of the prostate in the patients with prostate cancer: A systematic review and meta-analysis

**DOI:** 10.3389/fonc.2022.988490

**Published:** 2022-10-12

**Authors:** Yang Pan, Shangren Wang, Li Liu, Xiaoqiang Liu

**Affiliations:** Department of Urology, Tianjin Medical University General Hospital, Tianjin, China

**Keywords:** high-intensity focused ultrasound ablation, transurethral resection of prostate, prostatic neoplasms, treatment outcome, systematic review, meta-analysis

## Abstract

**Background:**

We aimed to conduct a systematic review and meta-analysis of studies reporting functional and oncologic outcomes of combining whole-gland high-intensity focused ultrasound ablation (HIFU) with transurethral resection of the prostate (TURP) in prostate cancer (PCa) patients.

**Methods:**

PubMed, Embase, Web of Science, Scopus, and Cochrane Library were systematically searched until June 30, 2022. The ROBINS-I tool scale was used to evaluate quality of eligible studies. Biochemical failure was defined according to the criteria used in each raw study. The presence of any cancer on follow-up biopsy was classified as “positive biopsy”. Patients able to penetrate their partner without pharmacologic support were rated potent. Meta-analysis was performed to evaluate functional outcomes using R project.

**Results:**

A total of 1861 patients in 15 eligible studies were included. All studies were identified as moderate or high quality. There were 1388 (74.6%) patients with low-risk or intermediate-risk PCa in 15 studies and 473 (25.4%) patients with high-risk PCa in 12 studies. The mean PSA nadir postoperatively ranged from 0.20 to 1.90 ng/mL within average time of 1.9-12 months. Biochemical failure rates in all 15 studies ranged from 6.3% to 34% within average time of 1.9-60 months. Eleven studies reported the rates of positive biopsy ranged from 3% to 29.7% within average time of 3-12 months postoperatively. Based on the results of single-arm meta-analysis, the pooled rates of any degree urinary incontinence, acute urinary retention, urinary tract infections, and urethral stricture were 9.4% (95% CI: 6.1%-12.6%), 0.9% (95% CI: 0%-2%), 2.6% (95% CI: 0.8%-4.3%), and 4.3% (95% CI: 1.4%-7.1%), respectively. The pooled rate of being potent after procedure in previously potent patients was 43.6% (95% CI: 27.3%-59.8%). The sensitivity analysis revealed all the pooled results was relatively reliable. Egger’s tests for the pooled results of acute urinary retention (*p* = 0.0651) and potency (*p* = 0.6749) both did not show significant publication bias.

**Conclusions:**

It appears that the combination treatment of whole-gland HIFU and TURP could be applied for PCa patients. It might have potential advantages of decreasing catheterization time and improving urinary status. Prospective and comparative studies are needed to validate our findings.

## Introduction

Prostate cancer (PCa) is a significant public health problem as the second most frequently diagnosed cancer in men. A systematic review of autopsy studies reported a prevalence of PCa at age < 30 years of 5% (95% CI: 3-8%), increasing by an odds ratio (OR) of 1.7 (1.6-1.8) per decade, to a prevalence of 59% (48-71%) by age > 79 years (1). Owing to the prostate-specific antigen (PSA) screening and many advanced imaging equipment such as multi-parametric magnetic resonance imaging (MRI), an increasing number of men have been diagnosed with localized PCa (2).

Based on the stage of disease and patients’ preference, localized PCa have usually been managed by active surveillance, radical prostatectomy or radiotherapy. However, there are some downsides to these treatments. On one hand, PCa patients may suffer from psychological stress like anxiety in the process of active surveillance; Meanwhile, the risk of disease progression is nonnegligible (3). On the other hand, radical prostatectomy or radiotherapy may result in postoperative complications such as erectile dysfunction, urinary incontinence, and gastrointestinal problems, the morbidity of which are 30-70%, 5-20%, and 5-10%, respectively (4, 5). Furthermore, some patients with old age, extreme obesity, or other severe cardiovascular diseases may not be suitable for radical prostatectomy or radiotherapy.

Among alternative therapies for PCa, high-intensity focused ultrasound ablation (HIFU) represents a promising technique in the middle ground between active surveillance and radical prostatectomy. Plenty of diseases such as uterine fibroids, liver tumors, and bone tumors can be successfully treated by HIFU and showed satisfactory efficacy and safety (6–8). In regards to urological diseases, HIFU has been increasingly used to manage localized PCa due to its acceptable efficacy and minimally invasive feature (9). Whole-gland HIFU is an effective and feasible treatment in PCa men and could be considered for patients unfit for radical surgery, reluctant to be under active surveillance, or willing a non-invasive treatment with a low morbidity burden.

When whole-gland HIFU is performed independently and not combined with other additional procedures, it has the following disadvantages, which may impact treatment outcomes. First, many HIFU devices have a relatively shorter focus length than the large prostate gland size. Prostates larger than 40 cc could not be completely ablated due to limited rectal movement space for the transrectal applicator, and limited HIFU penetration into the ventral areas and middle lobes. Second, most PCa patients may have calcifications or abscesses in the prostate. The calcifications or abscesses would disable ablative pulses from reaching the targeted focus and attenuate HIFU energy, which may cause a poor treatment outcome. Third, HIFU surgical time will be longer to focus and ablate the large prostate. Longer surgical time may have a potential impact on the safety, especially in patients with old age and general anesthesia. Fourth, the most common adverse events after whole-gland HIFU include the formation of bladder outlet obstruction or urethral stricture caused by edema or fibrosis on the prostatic urethra and bladder neck (10–14). Patients with urinary tract obstruction may feel unpleasant since urinary catheter need to be indwelled for a longer time to prevent acute urinary retention (AUR) or other serious complications. The catheterization may also be a frequent cause of urinary tract infections (UTIs), bladder stone, and similar other complications.

To improve treatment outcomes, some HIFU specialists advocate the method of combining HIFU with other additional procedures. Transurethral resection of the prostate (TURP) can remove partial tissues from the transitional zone of prostate gland. A meta-analysis including 20 contemporary trials with a maximum follow-up of five years revealed that TURP could result in a substantial mean Q-max improvement (+162%) and a significant reduction in the International Prostate Symptom Score (IPSS) assessment (-70%), quality-of-life (QoL) score (-69%), and postvoid residual (PVR) volume (-77%) (15). Therefore, the combination of HIFU and TURP are used to avoid the disadvantages of independent HIFU such as the risk of AUR.

Some studies reported that the combination of whole-gland HIFU and TURP could reduce the risk of prolonged catheterization significantly and improve posttreatment urinary status without additional morbidity (16–18). However, guidelines do not provide a definitive recommendation on whether to combine a TURP when applying whole-gland HIFU therapy for PCa patients. Furthermore, there was no study to systematically review the feasibility and efficacy of combining HIFU and TURP in PCa patients. Our goal is to conduct a systematic review and meta-analysis of studies evaluating the functional and oncologic outcomes of combining HIFU and TURP in patients with localized PCa.

## Methods

We performed a systematic review and meta-analysis following the Preferred Reporting Items for Systematic Reviews and Meta-analysis (PRISMA) guidelines. A protocol was submitted before the search and registered at the International Prospective Register of Systematic Reviews (PROSPERO, CRD42022332631). The research question was based on the PICOS (populations, interventions, comparisons, outcomes, and the study design) format, including population (men with PCa), intervention (HIFU and TURP in primary therapy), outcomes (oncologic and functional outcomes), and study design (randomized controlled trials, case series, prospective studies, retrospective series).

### Search strategy

Medline (via PubMed), Embase, Web of Science, Scopus, and Cochrane Library were searched for relevant articles from the inception of each database until June 30, 2022. The systematic searches included the following keywords: (“prostate cancer” OR “prostatic neoplasms”) AND (“high-intensity focused ultrasound ablation” OR “HIFU”) AND (“transurethral resection of prostate” OR “TURP”). The detailed search queries were combined with the corresponding items in each database (**Supplementary File 1**). All identified studies were then reviewed for eligibility. The reference lists and citations from key studies were also reviewed for additional eligible studies associated with our topic.

### Inclusion and exclusion criteria

The studies were included in this systematic review and meta-analysis if the following inclusion criteria were met: 1) study types: randomized controlled trials, case series, prospective studies, and retrospective studies; 2) studies included PCa patients who had undergone whole-gland HIFU therapy; 3) studies evaluated the functional and oncologic outcomes of combining HIFU and TURP treatment in patients with PCa; 4) studies provided sufficient data to calculate and analyze.

The exclusion criteria were as follows: 1) HIFU in salvage therapy; 2) non-whole gland HIFU therapy; 3) conference abstract; 4) guidelines; 5) review; 6) case report; 7) letter or comment paper; 8) animal studies; 9) image reports; 10) repeated publication.

### Data extraction and outcome measurement

All eligible articles and available data from the enrolled studies were extracted, respectively, by two independent reviewers and then checked by each other. If any disagreement appeared, a third reviewer would join in and discuss it with them to reach a consensus. Data were extracted from each study separately and outcome measures were set as follows: study region, study design, study duration, HIFU device, total sample size, age, PCa risk group according to D’Amico criteria, PSA levels before HIFU, prostate volume before HIFU, follow-up duration. Moreover, functional and oncologic outcomes after procedure were extracted and analyzed.

The primary endpoints were functional outcomes, including mean IPSS score change, postoperative catheterization time, urinary incontinence rate, rate of being potent, AUR rate, UTIs rate, and other complications rates. Partial urinary symptoms were evaluated by IPSS score (0-7 mildly symptomatic; 8-19 moderately symptomatic; 20-35 severely symptomatic). The change in mean IPSS score referred to the difference between postoperative and preoperative mean IPSS questionnaire scores. Patients able to penetrate their partner without pharmacologic support were rated potent. The rate of being potent referred to the proportion of potent patients after procedure in previously potent patients.

The secondary endpoints were oncologic outcomes, including PSA nadir, time to PSA nadir, biochemical failure rate (BCFR), BCFR in low-risk PCa patients, BCFR in intermediate-risk PCa patients, BCFR in high-risk PCa patients, time of calculating BCFR, reasons for prostate biopsy after HIFU, positive prostate biopsy rate, and time of calculating positive biopsy rate. Biochemical failure was defined according to the criteria used in each raw study, including: 1) the American society for therapeutic radiology and oncology (ASTRO) definition (three consecutive PSA increases after a nadir, with the date of failure being halfway between the nadir date and the first increase or any increase great enough to provoke the initiation of salvage therapy) (18); 2) the Phoenix definition (a rise ≥2 ng/mL above the nadir PSA) (19); and 3) the Stuttgart definition (an increase in PSA level of 1.2 ng/mL over the nadir) (20). Any cancer-positive biopsy sample after whole-gland HIFU treatment led to the classification of the considered patient in the “positive biopsy” result group.

### Quality assessment of included studies

The quality of included studies was assessed by two independent reviewers. The most precise tool to assess the quality of included articles is the risk of bias scales. If the study was a randomized controlled trial, the Cochrane risk of bias tool (RoB2) was used (21). For papers reporting on non-randomized controlled studies, the ROBINS-I (Risk of Bias in Non-randomized Studies of Interventions) tool was applied to assess the risk of bias (22). The ROBINS-I was used to assess the methodological quality of non-randomized studies on seven domains: confounding factors, selection of participants into the study, classification of interventions, deviations from intended interventions, missing data, measurement of outcomes, and selection of the reported results. Each domain was classified as having low, moderate, serious, critical, or no information available for risk of bias. The overall risk of bias for the study was determined by combining the levels of bias in each domain.

### Statistical analysis

Categorical variables were present as the number and proportion in the corresponding cohorts. Continuous variables were mainly present in the form of mean ± standard deviation; otherwise, continuous data were shown in the same form reported by the raw study. When reporting patients’ clinical characteristics, we regarded the total number of patients who performed the combination treatment of whole-gland HIFU and TURP as the denominator. As for the *de novo* complications such as urinary incontinence or erectile dysfunction, we regard the patients who had normal function preoperatively as the denominator.

All statistical analyses were performed and visualized using the software R version 4.1.2 (The R Foundation for Statistical Computing, MO, USA). Single-arm meta-analysis was performed to evaluate functional outcomes. The results are expressed as the pooled rate and 95% confidence interval (CI). The effect size of all pooled results was represented by 95% CI with an upper limit and a lower limit. The Cochrane Q chi-square test and I^2^ statistic were used to examine the heterogeneity across studies. The fixed-effects model was used for the pooled results with low heterogeneity (I^2^ ≤ 50%); otherwise, the random-effects model was used for analysis. The sensitivity analysis was performed by excluding each study one by one for the pooled results with high heterogeneity. Moreover, funnel plot and Egger’s test were used to detect the potential publication bias of included studies. Trim-and-fill analysis was performed if there was a potential publication bias. The value of *p* <.05 was considered statistically significant.

## Results

### Literature search

**Figure 1:**
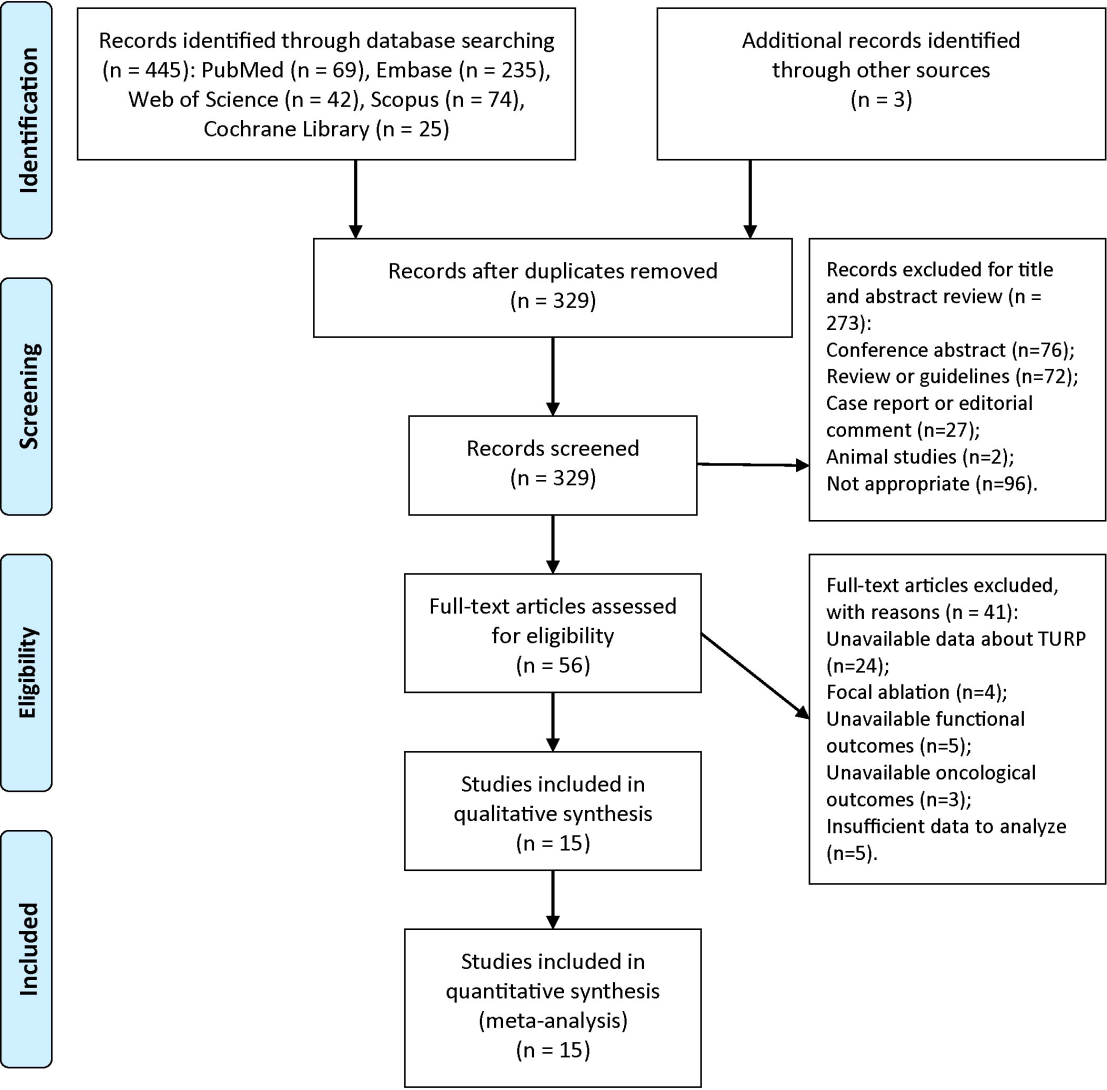
Flow diagram of identification and screening of eligible studies (PRISMA flow diagram).

A PRISMA flow chart of screening and selection results was shown in **Figure 1**. After searching databases systematically, we identified 445 potentially relevant articles. Three additional records were identified through other sources. There were 329 different articles after removing duplicates. According to the inclusion and exclusion criteria, 273 articles were excluded after reviewing their titles or abstracts. The remaining 56 studies were assessed for eligibility by reading full texts. After a full-text review, 15 eligible studies were included in this systematic review and meta-analysis finally (16–18, 23–34).

### Study characteristics

**Figure 2:**
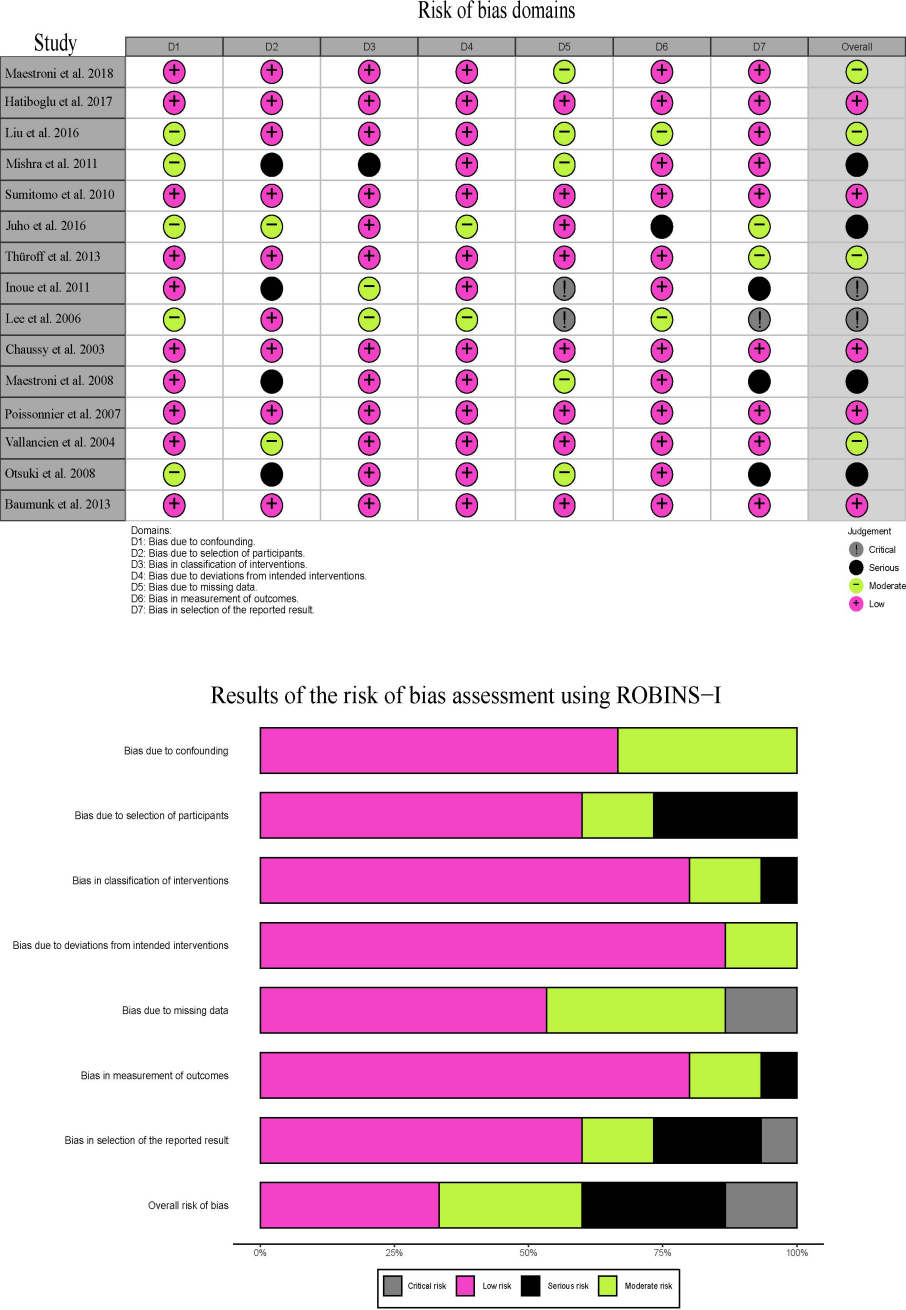
Quality assessment results of eligible studies using the ROBINS-I scales tool.

There were 5 prospective studies and 10 retrospective studies in this systematic review. Among these studies, only one was multicentric study and the remaining 14 studies were all single-institutional. Twelve of included studies utilized an Ablatherm^©^ device, and three studies utilized a Sonablate-500^©^ device. The characteristics of eligible studies are reported in **Table 1**. Quality of included studies was assessed using the ROBINS-I scales tool and the assessment results were shown in **Figure 2**.

**Table 1:** Characteristics and clinical data of eligible studies.

Author	Year	Study Region	Study design	Study duration	HIFU device	Sample Size	Age (years) ^a^	Prostate Cancer Risk Group(D’Amico criteria)	PSA Level before HIFU (ng/mL) ^a^	Prostate Volume before HIFU (mL) ^a^	Follow-up Duration (months) ^a^
Maestroni et al.	2018	Italy	Retro-, single-center	Apr. 2010-Dec. 2015	Ablatherm^©^	75	72.28 ± 4.63	LR 46 (61.4%), IR 19 (25.3%), HR 10 (13.3%)	9.44 ± 11.05	NR	29.9 (9-40) ^R^
Hatiboglu et al.	2017	Germany	Pros-, single-center	Feb. 2008-Dec. 2012	Ablatherm^©^	131	72.8 ± 6.0	LR 38 (29%), IR 77 (58.8%), HR 16 (12.2%)	9.6 ± 14.9	26.0 ± 12.5	22.2 ± 16.1
Liu et al.	2016	Taiwan	Retro-, single-center	Oct. 2008-Dec. 2013	Ablatherm^©^	120	68.06 ± 1.91	LR 15 (12.5%), IR 47 (39.2%), HR 58 (48.3%)	17.04 ± 21.88	21.97 ± 10.90	24.25 ± 11.15
Mishra et al.	2011	India	Retro-, single-center	Feb. 2008-Sep. 2010	Ablatherm^©^	24	70 (58-87) ^R^	LR 5 (21%), IR 4 (16%), HR 15 (62.5%)	NR	26.9 ± 8.5	10.4 (6-20) ^R^
Sumitomo et al.	2010	Japan	Pros-, multi-center	Apr. 2002-Mar. 2010	Sonablate-500^©^	64	69.0 ± 6.9	LR 18 (28.1%), IR 29 (45.3%), HR 17 (26.6%)	11.9 ± 7.2	19.9 ± 7.5	38.6 ± 15.3
Juho et al.	2016	Taiwan	Retro-, single-center	Oct. 2010-Mar. 2016	Ablatherm^©^	29	68.1 (59-82) ^R^	LR 11 (37.93%), IR 14 (48.27%), HR 4 (13.79%)	10.3 (0.5-31.5) ^R^	M 27.15 (9.32-59.6) ^R^	24.6
Thüroff et al.	2013	Germany	Pros-, single-center	1996-2009	Ablatherm^©^	704	68.4	LR 153 (21.6%), IR 270 (38.4%), HR 281 (40.0%)	9.9	21.5	M 144
Inoue et al.	2011	Japan	Retro-, single-center	May. 2003-Apr. 2010	Sonablate-500^©^	137	M 70 (50-82) ^R^	LR 29 (21%), IR 68 (50%), HR 40 (29%)	M 7.2 (2.8-100) ^R^	M 20 (8-52) ^R^	M 36 (12-84) ^R^
Lee et al.	2006	Korea	Retro-, single-center	Feb. 2004-Apr. 2005	Ablatherm^©^	58	70.0 ± 5.7	LR 13 (22.4%), IR 26 (44.8%), HR 19 (32.8%)	10.9 ± 6.4	36.6 ± 15.7	14 ± 4
Chaussy et al.	2003	Germany	Retro-, single-center	NR	Ablatherm^©^	175	68.4 ± 6.8	LR 71 (40.6%), IR 95 (54.3%), HR 9 (5.1%)	8.0 ± 3.4	20.5 ± 9.8	10.9 ± 6.2
Maestroni et al.	2008	Italy	Pros-, single-center	May. 2006-Apr. 2007	Ablatherm^©^	25	71.6 (56-78) ^R^	LR 17 (68%), IR 6 (24%), HR 2 (8%)	9.7 (0.78-54.9) ^R^	NR	6
Poissonnier et al.	2007	France	Retro-, single-center	1993-2003	Ablatherm^©^	227	68.8 ± 5.82	LR 152 (67%), IR 75 (33%)	6.99 ± 3.48	23.9 ± 10.26	27.5 ± 20
Vallancien et al.	2004	France	Retro-, single-center	Apr. 1999-Nov. 2001	Ablatherm^©^	30	72 (61-79) ^R^	LR or IR 30 (100%)	7 (1-10) ^R^	30 (11-45) ^R^	M 20 (3-38) ^R^
Otsuki et al.	2008	Japan	Retro-, single-center	Apr. 2015-Aug. 2006	Sonablate-500^©^	18	66 (58-74) ^R^	LR 13 (72.2%), IR 3 (16.7%), HR 2 (11.1%)	M 2.7 (0.17-12.0) ^R^	M 22.0 (10.0-32.2) ^R^	M 10 (5-15) ^R^
Baumunk et al.	2013	Germany	Pros-, single-center	2005-2009	Ablatherm^©^	44	70.89 ± 4.29	LR 44 (100%)	5.41 ± 2.6	20.81 ± 18.85	M 27.12

TURP, Transurethral resection of the prostate; HIFU, High-intensity focused ultrasound ablation; PSA, Prostate-specific antigen; Retro-, Retrospective; Pros-, Prospective; M, Median; R, Range; LR, Low-risk; IR, Intermediate-risk; HR, High-risk; NR, Not reported.

a Continuous variables are mainly presented in the form of mean or mean ± standard deviation; otherwise, continuous data are shown in the same form reported by the raw studies.

### Clinical data of eligible patients

A total of 1861 patients in 15 studies were included in this systematic review and meta-analysis eventually. The average age ranged from 64.8 to 72.8 years old. Thirteen studies reported the prostate volume before HIFU, and the mean volume ranged from 19.9 to 36.6 cc. The mean PSA levels before HIFU ranged from 5.41 to 17.04 ng/mL in 14 studies. Ten studies showed an average preoperative PSA value of less than 10 ng/mL. According to D’Amico criteria, 1388 (74.6%) patients with low-risk or intermediate-risk PCa in 15 studies and 473 (25.4%) patients with high-risk PCa in 12 studies were treated. All 15 studies reported the follow-up duration and the average ranged from 6 to 38.6 months. Demographic and clinical characteristics of patients in the included studies were shown in **Table 1**.

### Oncologic outcomes

Oncologic outcomes were not pooled because the definition criteria and checking time points showed significant variations among different studies. The PSA nadir and the time to PSA nadir after procedure were reported in all 15 studies. The mean PSA nadir ranged from 0.20 to 1.90 ng/mL with the average time to PSA nadir ranging from 1.9 to 12 months. Biochemical failure was defined according to the criteria used in each raw study. Biochemical failure rates (BCFR) in all 15 studies ranged from 6.3% to 34%, with the average time of calculating BCFR ranging from 1.9 to 60 months postoperatively. Furthermore, the ranges of BCFR in low-risk, intermediate-risk, and high-risk PCa patients were 0-30%, 0-33.3%, and 0-100%, respectively. Many studies mentioned that prostate biopsy after HIFU was performed due to a routine follow-up biopsy or a rising PSA level. The presence of any cancer on biopsy was classified as “positive biopsy”. As reported in 11 studies, the rate of positive biopsy ranged from 3% to 29.7%, with the average time of calculating positive biopsy rate ranging from 3 to 12 months postoperatively. The important oncologic outcomes of eligible studies are listed in **Table 2**.

**Table 2:** Oncological outcomes of eligible studies.

Study	Sample Size	PSA Nadir (ng/mL) ^a^	Time to PSA Nadir (months po.) ^a^	BCFR	BCFR in LR Patients	BCFR in IR Patients	BCFR in HR Patients	Time of BCFR (months po.)	BCF Definition Criteria	Positive Prostate Biopsies Rate ^b^	Time of Positive Biopsy (months po.)	Reasons for Prostate Biopsy after Operation
Maestroni et al., 2018	75	1.19 (0.065-25.3) ^R^	3.0 ± 2.3	13/75 (17.3%)	4/46 (6.25%)	2/19 (10.53%)	7/10 (70%)	12.5 (3-40) ^R^	Phoenix	7/45 (15.5%)	NR	FUB 6m po. or Rising PSA
Hatiboglu et al., 2017	131	0.6 ± 1.2	4.7 ± 5.7	28/131 (21.4%)	10/38 (26.3%)	12/77 (15.6%)	6/16 (3.75%)	15.5 ± 11.6	Stuttgart	3/28 (10.7%)	6	FUB 6m po.
Liu et al., 2016	120	0.64 ± 1.77	3.41 ± 3.83	22/120 (18.3%)	1/15 (6.7%)	4/47 (8.5%)	17/58 (29.3%)	21.34 ± 11.22	Phoenix	NR	NR	Rising PSA
Mishra et al., 2011	24	0.53, M 0.3	6.0 ± 3.0	2/24 (8.3%)	0/5 (0%)	0/4 (0%)	2/15 (13.3%)	7/9	Phoenix	2/8 (25%)	7/9	NR
Sumitomo et al., 2010	64	0.323 ± 1.287	12.0	4/64 (6.3%)	NR	NR	NR	12	Phoenix	9/53 (17%)	NR	FUB
Juho et al., 2016	29	0.2116	1.9	6/29 (20.7%)	0/11 (0%)	1/14 (7.1%)	2/4 (50%)	1.9	Stuttgart	1/5 (20%)	6	BCFR developed
Thüroff et al., 2013	704	1.7, M 0.1	M 2.1	141/704 (20%)	21/153 (13.7%)	59/270 (21.9%)	61/281 (21.75%)	24	Phoenix	NR	NR	NR
Inoue et al., 2011	137	M 0.07 (0.01-2.01) ^R^	M 2 (1-6) ^R^	16.4%	3.3%	16.1%	26.5%	36	Phoenix	4/133 (3%)	6	FUB 6m po.
Lee et al., 2006	58	0.2 (0.01-7.60) ^R^	2.2	18/58 (31%)	2/13 (15.4%)	6/26 (23.1%)	10/19 (52.6%)	14	ASTRO	10/58 (17.2%)	M 10 (3-14) ^R^	Rising PSA
Chaussy et al., 2003	175	0.26 ± 0.90	3.5	35/175 (20%)	NR	NR	NR	26.9	ASTRO	52/175 (29.7%)	12	FUB 12m po.
Maestroni et al., 2008	25	0.832	3.0	4/25 (16%)	1/17 (5.9%)	1/6 (16.7%)	2/2 (100%)	NR	ASTRO	3/25 (12%)	6	FUB 6m po.
Poissonnier et al., 2007	227	0.33 ± 0.70	4.4	77/227 (34%)	NR	NR	NR	60	ASTRO	31/227 (13.7%)	3	FUB 3m po. or Rising PSA
Vallancien et al., 2004	30	0.9 (0.0-2.6) ^R^	M 12.0	4/30 (13.3%)	NR	NR	NR	NA	ASTRO	5/30 (16.7%)	12	FUB 12m po. or Rising PSA
Otsuki et al., 2008	18	M 0.43	M 3.0	2/18 (11.1%)	1/13 (7.7%)	1/3 (33.3%)	0/2 (0%)	6	ASTRO	NR	NR	NR
Baumunk et al., 2013	44	0.246 ± 0.59	10.47 ± 11.04	13/44 (30%)	13/44 (30%)	NR	NR	48	Phoenix	NR	NR	NR

HIFU, High-intensity focused ultrasound ablation; PSA, Prostate-specific antigen; IQR, Interquartile range; M, median; R, range; BCFR, Biochemical failure rate; BCF, Biochemical failure; LR, Low-risk; IR, Intermediate-risk; HR, High-risk; ASTRO, American society for therapeutic radiology and oncology; FUB, Follow-up biopsy; Po., Post operation; 6m po., 6 months post operation; NR, not reported.

a Continuous variables are mainly presented in the form of mean or mean ± standard deviation; otherwise, continuous data are shown in the same form reported by the raw studies.

b Positive prostate biopsies patients/total patients who had undergone prostate biopsies after the procedure.

### IPSS score change and postoperative catheterization time

Eleven studies reported the change in mean IPSS scores, which referred to the difference between postoperative and preoperative values. The change in mean IPSS score ranged from (-4.8) score to (+2.08) score. Ten studies reported a decreasing average IPSS score than that of pre-procedure, while one study (28) reported an increasing IPSS score after the operation. The postoperative catheterization time was reported in ten studies, and the average ranged from 3.9 to 15 days. The important functional outcomes of eligible studies are displayed in **Table 3**.

**Table 3:** Functional outcomes of eligible studies.

Study	Sample Size	IPSS Change	Catheterization Time (days) ^a^	UI Rate	Rate of Being Potent ^b^	AUR Rate	UTIs Rate	Urethral Stricture Rate	Other Complications Rate
Maestroni et al., 2018	75	-3.46	9.3 ± 4.5	13/75 (17.3%)	4/16 (25%)	0%	0%	0%	Recto-vesical fistula (1.3%)
Hatiboglu et al., 2017	131	-1.7	14.5 ± 15.0	22/93 (23.7%)	36/101 (35.6%)	0%	4/131 (3.1%)	0%	Infravesical obstruction (22.2%)
Liu et al., 2016	120	-2.9	4.12 ± 2.70	3/120 (2.5%)	11/32 (34.4%)	0%	0%	13/120 (10.8%)	Epididymitis (5.8%)
Mishra et al., 2011	24	-4.8	3.9	2/24 (8.3%)	0/13 (0%)	0%	5/24 (20.8%)	2/24 (8.3%)	Secondary hemorrhage (3.3%)
Sumitomo et al., 2010	64	-2.2	6.0 ± 3.5	2/64 (3.1%)	13/43 (30.2%)	5/64 (7.8%)	0%	7/64 (10.9%)	RUF (0.8%), BNC (1.6%), Epidydimitis (0.8%)
Juho et al., 2016	29	NR	7.0	5/29 (17.2%)	22/29 (75.9%)	0%	6/29 (20.6%)	8/29 (27.58%)	BNC (24.1%), BOO (48.3%)
Thüroff et al., 2013	704	NR	NR	28/704 (4%)	NR	32/704 (4.6%)	15/704 (2.1%)	0%	RUF (0.2%), Perineal pain (0.7%)
Inoue et al., 2011	137	NR	NR	16/137 (11.7%)	37/59 (63%)	0%	6/137 (4.4%)	10/137 (7.2%)	Difficult voiding (22.3%), Epididymitis (2.7%)
Lee et al., 2006	58	NR	15 (3-43) ^R^	9/58 (16%)	NR	2/58 (3.45%)	0%	4/58 (6.9%)	Delayed passage of necrotic debris (14%)
Chaussy et al., 2003	175	-3.32	13.7 ± 16.6	12/175 (6.9%)	119/175 (68%)	0%	20/175 (11.4%)	0%	No other complications
Maestroni et al., 2008	25	-3.2	10.4 (1-45) ^R^	3/25 (12%)	0/3 (0%)	2/25 (8%)	0%	0%	Perineal pain (20%), Recto-vesical fistula (4%)
Poissonnier et al., 2007	227	NR	7.0	30/227 (13%)	25/41 (61%)	0%	4/227 (2%)	27/227 (12%)	Sloughing (9%), Urgency (5%), Perineal pain (3%)
Vallancien et al., 2004	30	-0.8	M 2	1/30 (3.3%)	11/14 (78.6%)	2/30 (6.7%)	3/30 (10%)	0%	Hematuria (66%)
Otsuki et al., 2008	18	-4.0	5.67 ± 3.17	2/18 (11.1%)	NR	0%	0%	1/18 (5.6%)	No other complications
Baumunk et al., 2013	44	+2.08	NR	3/44 (6.8%)	NR	1/44 (2.3%)	2/44 (4.5%)	0%	No other complications

TURP, Transurethral resection of the prostate; HIFU, High-intensity focused ultrasound ablation; IPSS, the International Prostate Symptom Score; M, Median; R, Range;

NR, Not reported; UI, Urinary incontinence; AUR, Acute urine retention; UTIs, Urinary tract infections; RUF, Recto-urethral fistula; BNC, Bladder neck contracture; BOO, Bladder outlet obstruction.

a Continuous variables are mainly presented in the form of mean ± standard deviation; otherwise, continuous data are shown in the same form reported by the raw studies.

b Rate of being potent after/before HIFU. Patients able to penetrate their partner without pharmacologic support were rated potent.

### Urinary incontinence

**Figure 3:**
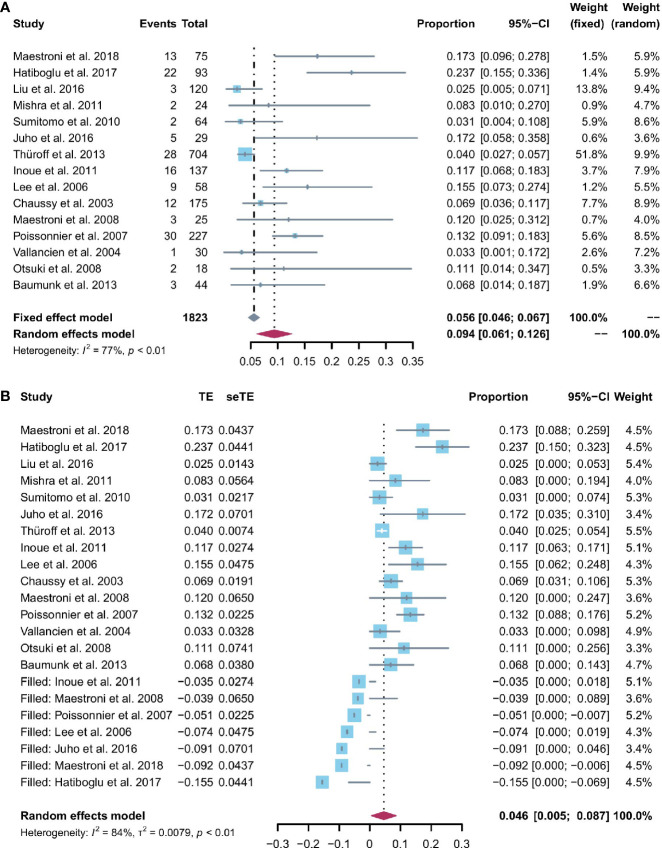
**(A)** Forest plot of the pooled results of any degree urinary incontinence; **(B)** Forest plot of the pooled results of any degree urinary incontinence after trim-and-fill analysis.

All 15 studies reported the incidence of any degree urinary incontinence after the operation. Based on a sing-arm meta-analysis of 15 studies, the pooled rate of urinary incontinence was 9.4% (95% CI: 6.1% to 12.6%; I^2^ = 77%; *p* < 0.01; **Figure 3A**). The sensitivity analysis of the pooled results of urinary incontinence was reliable (**Figure S1**). Based on the funnel plot (**Figure 6A**) and Egger’s test (*p* = 0.0046), assessment results of the pooled rate of urinary incontinence showed a potential publication bias among included studies. Trim-and-fill analysis estimated 7 missing studies (**Figure 3B**). The pooled rate of urinary incontinence based on this analysis was 4.6% (95% CI: 0.5% to 8.7%; **Figure 3B**), which was lower than the originally reported pooled rate. After trim-and-fill analysis, the funnel plot (**Figure 6B**) and Egger’s test (*p* = 0.7914) did not show significant publication bias.

### Potency

**Figure 4:**
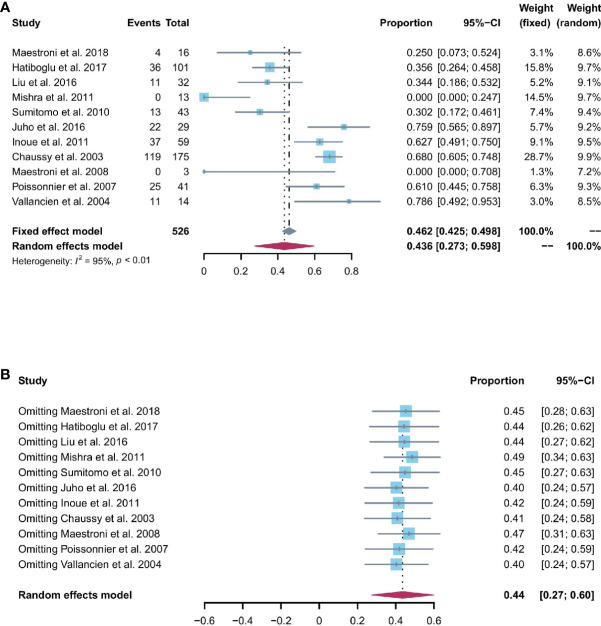
**(A)** Forest plot of the pooled results of being potent; **(B)** The sensitivity analysis of the pooled results of being potent.

Patients able to penetrate their partner without pharmacologic support were rated potent. Eleven studies reported the cases of being potent after and before the operation. Based on a sing-arm meta-analysis of 11 studies, the pooled rate of being potent was 43.6% (95% CI: 27.3% to 59.8%; I^2^ = 95%; *p* < 0.01; **Figure 4A**). The sensitivity analysis of the pooled results of being potent was reliable (**Figure 4B**). Based on the funnel plot (**Figure 6C**) and Egger’s test (*p* = 0.6749), assessment results of the pooled rate of being potent did not show a publication bias among included studies.

### Acute urinary retention

**Figure 5:**
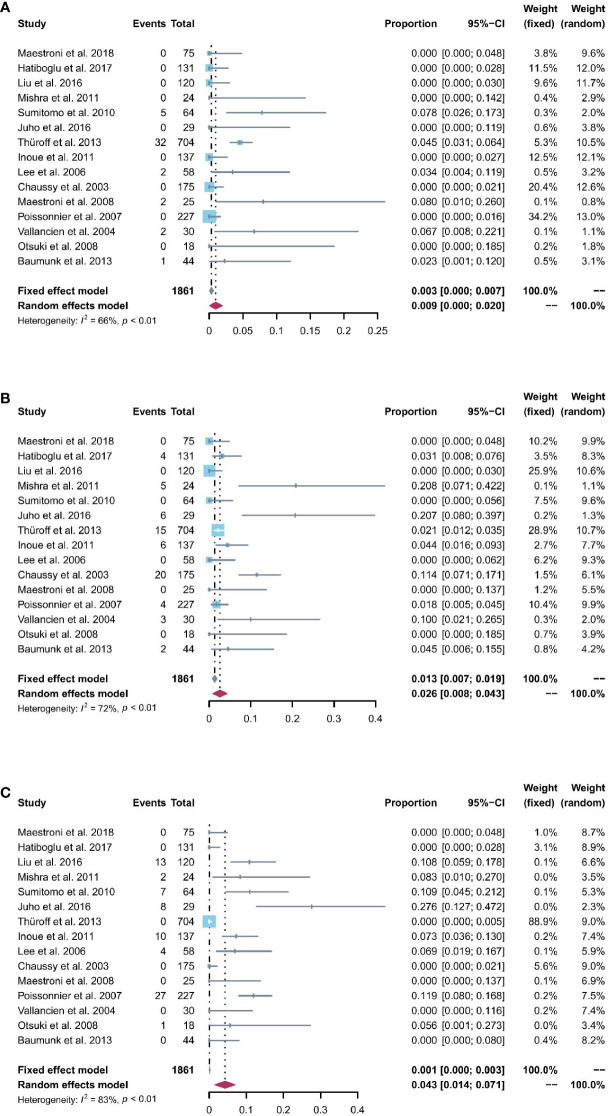
**(A)** Forest plot of the pooled results of acute urinary retention; **(B)** Forest plot of the pooled results of urinary tract infections; **(C)** Forest plot of the pooled results of urethral stricture.

All 15 studies reported the incidence of AUR after the operation. The pooled rate of AUR was 0.9% (95% CI: 0% to 2%; I^2^ = 66%; *p* < 0.01; **Figure 5A**). The sensitivity analysis (**Figure S2**) showed that the pooled results of AUR changed significantly after omitting the study by Thüroff2013 (27). The funnel plot (**Figure S3**) showed a possibly potential publication bias; however, the result of Egger’s test (*p* = 0.0651) was not statistically significant.

### Urinary tract infections

The pooled rate of UTIs from 15 studies was 2.6% (95% CI: 0.8% to 4.3%; I^2^ = 72%; *p* < 0.01; **Figure 5B**). The sensitivity analysis of the pooled results of UTIs was relatively reliable (**Figure S4**). Based on the funnel plot (**Figure S5**) and Egger’s test (*p* = 0.024), assessment results of the pooled rate of UTIs showed a potential publication bias among included studies. Trim-and-fill analysis estimated 4 missing studies (**Figure S6**). The pooled rate of UTIs based on this analysis was 1.3% (95% CI: 0 to 4.1%; **Figure S6**), which was lower than the originally reported pooled rate. After trim-and-fill analysis, the funnel plot (**Figure S7**) and Egger’s test (*p* = 0.8616) did not show significant publication bias.

### Urethral stricture

**Figure 6:**
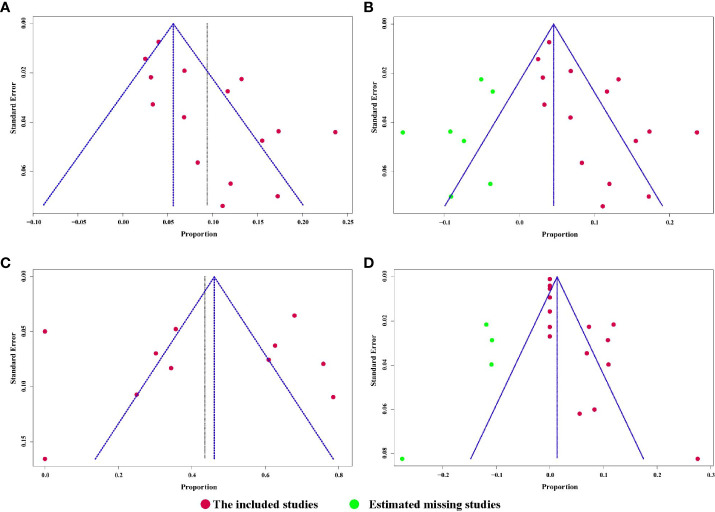
**(A)** Funnel plot of the pooled results of urinary incontinence.; **(B)** Funnel plot of the pooled results of urinary incontinence after trim-and-fill analysis; **(C)** Funnel plot of the pooled results of being potent; **(D)** Funnel plot of the pooled results of urethral stricture after trim-and-fill analysis.

The pooled rate of urethral stricture from 15 studies was 4.3% (95% CI: 1.4% to 7.1%; I^2^ = 83%; *p* < 0.01; **Figure 5C**). The sensitivity analysis of the pooled results of urethral stricture was relatively reliable (**Figure S8**). Based on the funnel plot (**Figure S9**) and Egger’s test (*p* = 0.0033), assessment results of the pooled rate of urethral stricture showed a potential publication bias among included studies. Trim-and-fill analysis estimated 4 missing studies (**Figure S10**). The pooled rate of urethral stricture based on this analysis was 1.4% (95% CI: 0 to 5.5%; **Figure S10**), which was lower than the originally reported pooled rate. After trim-and-fill analysis, the funnel plot (**Figure 6D**) and Egger’s test (*p* = 0.5348) did not show significant publication.

## Discussion

The present study was the first systematic review and meta-analysis on reporting functional and oncologic outcomes of the combination of whole-gland HIFU and TURP in localized PCa patients. Our systematic review summarized all kinds of baseline characteristics, functional outcomes, and oncologic outcomes in eligible studies relevant to combining whole-gland HIFU with TURP to manage PCa cases. Furthermore, functional outcomes after the combination treatment of whole-gland HIFU and TURP were well pooled and evaluated based on the results of meta-analysis. Our work might be significant to clarify the outcomes of combining whole-gland HIFU with TURP, especially for clinicians who prepare to carry out the whole-gland HIFU operation in PCa patients.

Over the past time, the surgical technique of performing the operation and the instrumental equipment have undergone improvement. The whole-gland HIFU has its unique advantages compared to other whole-gland treatments like radical surgery and radiotherapy. First, whole-gland HIFU is a feasible treatment in elderly men with PCa and could be considered for patients either unfit for radical surgery, or willing a non-invasive treatment with a low morbidity burden. Second, whole-gland HIFU therapy is an effective treatment option with a low risk of side effects. It could be considered a reasonable choice for carefully selected patients with localized PCa, reluctant to be under active surveillance. One of the advantages of HIFU therapy over active surveillance is lower anxiety among treated patients. Third, there is already evidence that the chances for overall survival and metastasis-free survival up to 5 years are the same for both patients who have undergone whole-gland HIFU treatment and radical prostatectomy (35). Moreover, whole-gland HIFU could achieve good long-term cancer control up to 21 years in low- and intermediate-risk PCa patients (36). Therefore, whole-gland HIFU might still be a desirable therapeutic option for PCa patients.

One of common adverse events after whole-gland HIFU is the formation of bladder outlet obstruction or urethral stricture caused by edema or fibrosis of the prostatic urethra and bladder neck. Verde et al. reported the oncologic and functional outcomes of whole-gland HIFU as first-line treatment for localized PCa patients between January 2005 and July 2018; they found that symptoms related to bladder outlet obstruction were the most frequently recorded adverse events (12). Dosanjh et al. studied the patients undergoing HIFU for prostate cancer between April 2007 and March 2018 in an English national database (Hospital Episode Statistics); they found that 10.3% of patients developed urethral stricture following HIFU (14). Byun et al. also retrospectively investigated patients who underwent HIFU for localized PCa between 2018 and 2020; they found that 20.9% of patients required additional endoscopic surgery for bladder outlet obstruction (13). Based on these previous reports, we consider that the problem of bladder outlet obstruction or urethral stricture after HIFU might still persist in recent years. However, the pooled rates of AUR and urethral stricture after the combination treatment of whole-gland HIFU and TURP were 0.9% and 4.3%, respectively. Therefore, the combination of HIFU and TURP could significantly reduce the risk of AUR and urethral stricture.

Sumitomo et al. reported that the AUR rate postoperatively was 10.9% when whole-gland HIFU was performed solely; however, the rate significantly decreased to 3.9% when combined with TURP (31). Chaussy et al. found that mean postoperative urinary catheter time in the HIFU and TURP group (13.7 days) was significantly shorter than that in the sole HIFU group (45.1 days) (16). Horiuchi et al. also showed that the rate of urinary retention due to urethral stricture changed from 13.3% to 0 when the combination of whole-gland HIFU and enucleation of the prostate were applied for localized PCa (37). Based on these previous studies, we considered that the combination treatment of whole-gland HIFU and TURP might develop a better postoperative urinary status than the sole HIFU procedure.

The pooled rates of urinary incontinence and potency after the combination treatment of whole-gland HIFU and TURP were 9.4% and 43.6%, respectively. Some previous studies reported the rates of urinary incontinence and potency after whole-gland HIFU treatment without combining a TURP. Blana et al. showed that 34.3% of PCa patients presented with at least one episode of incontinence, and 56.8% in previously potent patients claimed sexual potency after HIFU (38). Mearini et al. conducted a prospective trial with long-term follow-up, which reported that 16% of PCa patients developed urinary incontinence and 44% of the preoperative potent patients were still potent after the sole whole-gland HIFU treatment (39). Therefore, the combination treatment of whole-gland HIFU and TURP seems not increase the rates of urinary incontinence and potency significantly.

It is relatively difficult to evaluate the oncologic outcomes after the procedure. On one hand, PSA is expected to decrease after the whole-gland treatment like radical prostatectomy. However, it is uncertain that PSA levels after HIFU and TURP treatments can be used to evaluate the recurrence of PCa accurately because the PSA level at this moment is a reflection of combining inflammation, remaining prostate tissue, and malignancy. On the other hand, prostate biopsy is regarded as a better method to evaluate the cancer control in the short term. However, it is challenging to avoid the biopsy targeting and sampling mistakes, which are related to the experience and techniques of a surgeon. Furthermore, the definition criteria of PCa recurrence and the time points of checking PSA levels or undergoing prostate biopsy also showed significant variations among different studies. In recent years, even though serum and urinary biomarkers or genomic tests could be regarded as the short-term endpoint for PCa recurrence, the impact of combining HIFU with TURP therapy for PCa on these biomarkers keeps unknown. Translational researches are required to verify the function of these biomarkers or genomic tests after the operation. In the future, a combination of imaging, PSA levels, biomarkers, genomic tests, and prostate biopsy will be extremely helpful for better drawing up the follow-up strategies after the procedure for PCa.

Some limitations might exist in this study. First, 12 raw studies used an Ablatherm device, while 3 raw studies used a Sonablate-500 device, thus there could be a bias caused by the different systems. Second, almost 75% of the patients had a low-risk or intermediate-risk prostate cancer, while 25% had a high-risk prostate cancer, which might represent a difference in the assessment of oncological outcomes. These factors led to the heterogeneity of outcomes to a certain extent, which might also be a limit for this study. Despite these limitations, this systematic review and meta-analysis provides valuable evidence and reference for treatment outcomes of the combination of whole-gland HIFU and TURP in PCa patients.

## Conclusion

It appears that the combination treatment of whole-gland HIFU and TURP could show satisfactory functional outcomes in PCa patients. The combination of whole-gland HIFU with TURP treatment might have potential advantages of reducing prostate volume, decreasing postoperative catheterization time, and improving postoperative urinary status. Prospective and comparative studies with long follow-up duration are needed to generate reliable evidence and validate long-term oncologic and functional outcomes of whole-gland HIFU and TURP in PCa patients.

## Data availability statement

All datasets presented in this study are included in the article/**Supplementary Material**.

## Author contributions

Project development: YP and XL. Data collection or management: YP, SW, and LL. Data analysis: YP, and SW. Manuscript writing/editing: YP and XL. All authors contributed to the article and approved the submitted version.

## Funding

Our project was supported by National Natural Science Foundation of China (No. 82171594). The role of the funders was in the design and writing the study.

## Conflict of interest

The authors declare that the research was conducted in the absence of any commercial or financial relationships that could be construed as a potential conflict of interest.

## Publisher’s note

All claims expressed in this article are solely those of the authors and do not necessarily represent those of their affiliated organizations, or those of the publisher, the editors and the reviewers. Any product that may be evaluated in this article, or claim that may be made by its manufacturer, is not guaranteed or endorsed by the publisher.
