# Supplementary material for: Whole-gland high-intensity focused ultrasound ablation and transurethral resection of the prostate in the patients with prostate cancer: A systematic review and meta-analysis
Source: Front Oncol. 2022 Oct 12;12:988490. doi: 10.3389/fonc.2022.988490 (PMC9597449; doi:10.3389/fonc.2022.988490)

Supplementary File 2

Manuscript Title: Whole-gland High-Intensity Focused Ultrasound Ablation and Transurethral Resection of the Prostate in the Patients with Prostate Cancer: A Systematic Review and Meta-Analysis

The supplementary file 2 contains 10 figures (Figure S1-S10).

Figure S1. The sensitivity analysis of the pooled results of urinary incontinence.

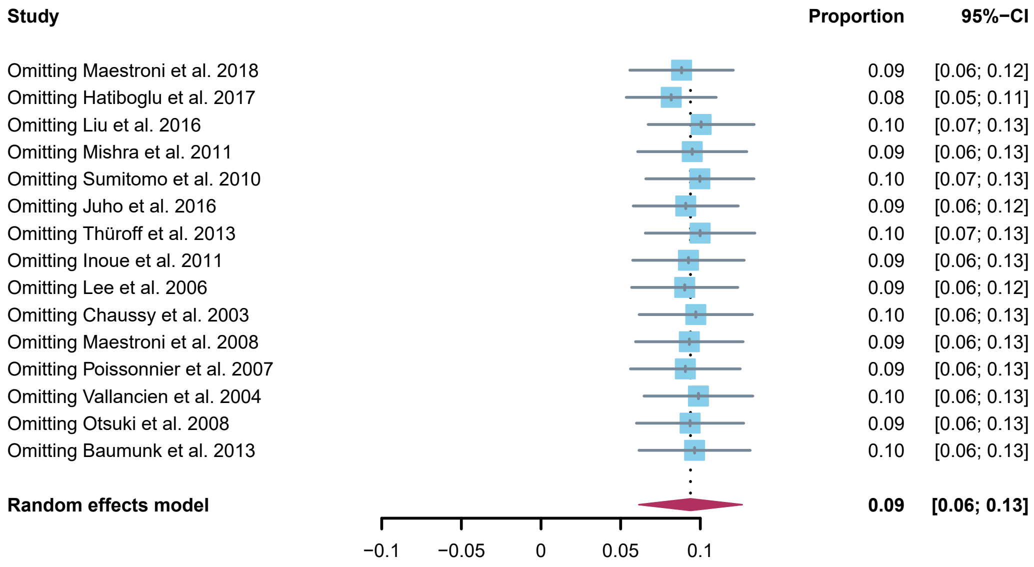

Figure S2. The sensitivity analysis of the pooled results of acute urinary retention.

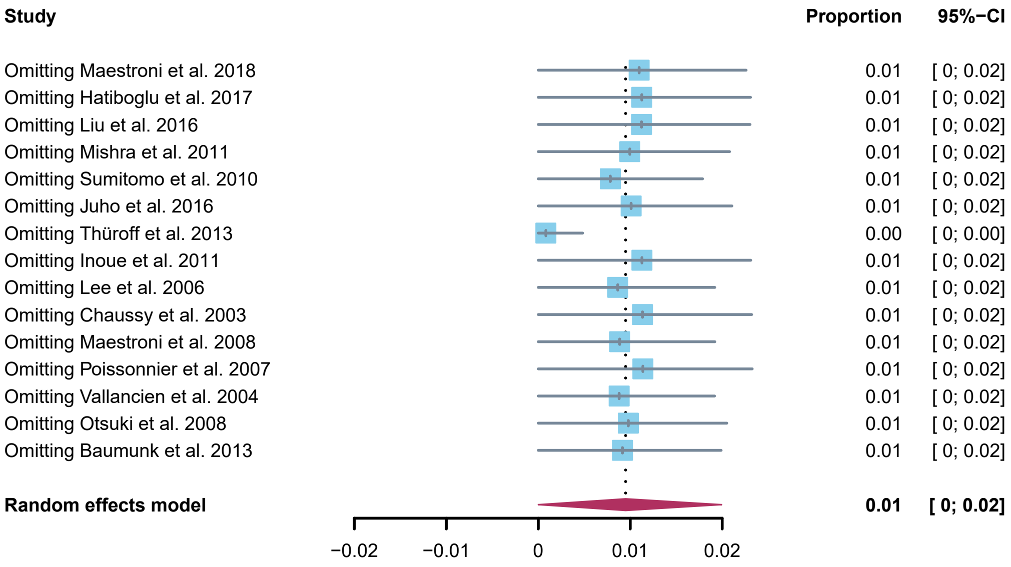

Figure S3. The funnel plot of the pooled results of acute urinary retention.

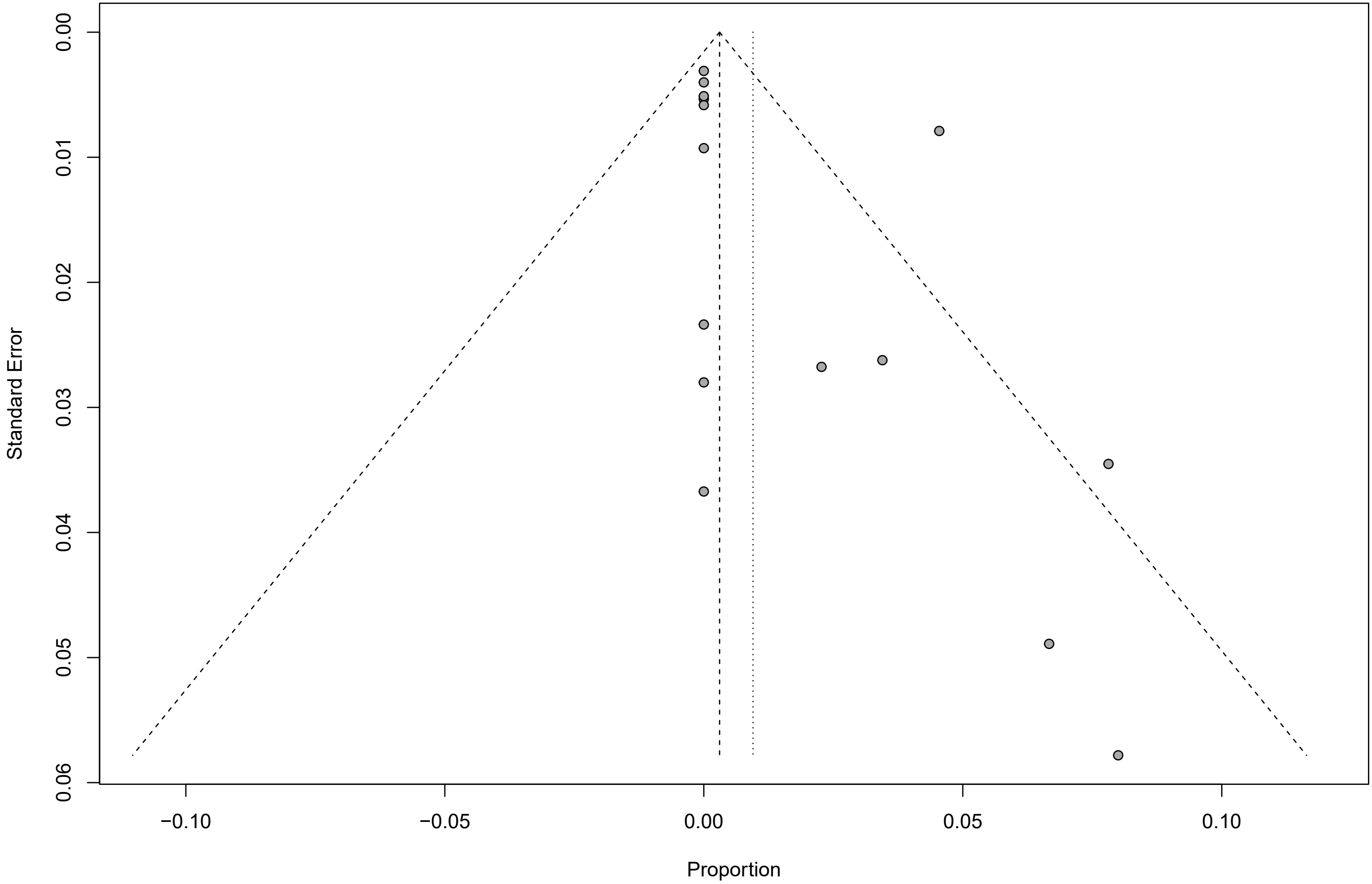

Figure S4. The sensitivity analysis of the pooled results of urinary tract infections.

Study	Proportion	95%−CI
Omitting Maestroni et al. 2018	0.03	[0.01; 0.05]
Omitting Hatiboglu et al. 2017	0.03	[0.01; 0.05]
Omitting Liu et al. 2016	0.03	[0.01; 0.05]
Omitting Mishra et al. 2011	0.02	[0.01; 0.04]
Omitting Sumitomo et al. 2010	0.03	[0.01; 0.05]
Omitting Juho et al. 2016	0.02	[0.01; 0.04]
Omitting Thüroff et al. 2013	0.03	[0.01; 0.05]
Omitting Inoue et al. 2011	0.02	[0.01; 0.04]
Omitting Lee et al. 2006	0.03	[0.01; 0.05]
Omitting Chaussy et al. 2003	0.01	[0.00; 0.02]
Omitting Maestroni et al. 2008	0.03	[0.01; 0.05]
Omitting Poissonnier et al. 2007	0.03	[0.01; 0.05]
Omitting Vallancien et al. 2004	0.02	[0.01; 0.04]
Omitting Otsuki et al. 2008	0.03	[0.01; 0.05]
Omitting Baumunk et al. 2013	0.03	[0.01; 0.04]
**Random effects model**	**0.03**	**[0.01; 0.04]**

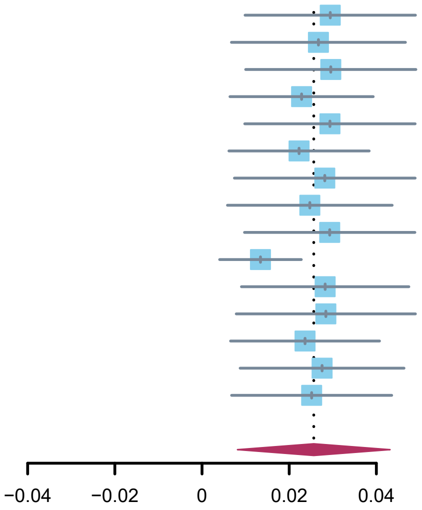

Figure S5. The funnel plot of the pooled results of urinary tract infections.

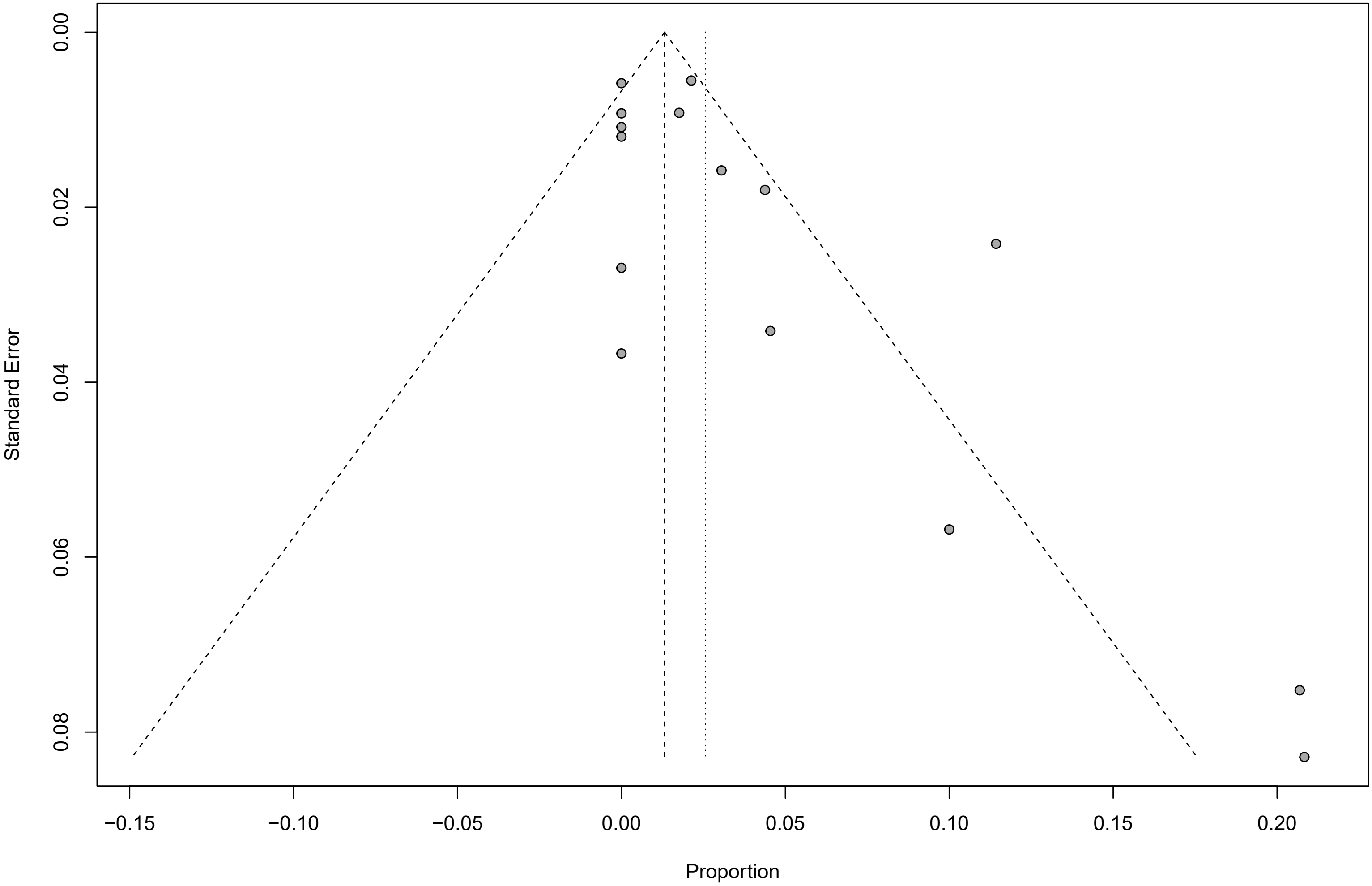

Figure S6. The forest plot of the pooled results of urinary tract infections after trim-and-fill analysis.

Study	TE	seTE	Proportion	95%−CI	Weight
Maestroni et al. 2018	0.000	0.0093	0.000	[0.000; 0.018]	7.1%
Hatiboglu et al. 2017	0.031	0.0158	0.031	[0.000; 0.061]	6.8%
Liu et al. 2016	0.000	0.0058	0.000	[0.000; 0.011]	7.3%
Mishra et al. 2011	0.208	0.0828	0.208	[0.046; 0.371]	2.1%
Sumitomo et al. 2010	0.000	0.0108	0.000	[0.000; 0.021]	7.1%
Juho et al. 2016	0.207	0.0752	0.207	[0.059; 0.354]	2.4%
Thüroff et al. 2013	0.021	0.0055	0.021	[0.010; 0.032]	7.3%
Inoue et al. 2011	0.044	0.0180	0.044	[0.008; 0.079]	6.6%
Lee et al. 2006	0.000	0.0119	0.000	[0.000; 0.023]	7.0%
Chaussy et al. 2003	0.114	0.0242	0.114	[0.067; 0.162]	6.1%
Maestroni et al. 2008	0.000	0.0269	0.000	[0.000; 0.053]	5.8%
Poissonnier et al. 2007	0.018	0.0092	0.018	[0.000; 0.036]	7.1%
Vallancien et al. 2004	0.100	0.0568	0.100	[0.000; 0.211]	3.4%
Otsuki et al. 2008	0.000	0.0367	0.000	[0.000; 0.072]	4.9%
Baumunk et al. 2013	0.045	0.0341	0.045	[0.000; 0.112]	5.2%
Filled: Vallancien et al. 2004	−0.078	0.0568	−0.078	[0.000; 0.033]	3.4%
Filled: Chaussy et al. 2003	−0.093	0.0242	−0.093	[0.000; −0.045]	6.1%
Filled: Juho et al. 2016	−0.185	0.0752	−0.185	[0.000; −0.038]	2.4%
Filled: Mishra et al. 2011	−0.187	0.0828	−0.187	[0.000; −0.024]	2.1%
**Random effects model**			**0.013**	**[0.000; 0.041]**	**100.0%**

Heterogeneity: $I^2 = 78\%$, $p < 0.01$

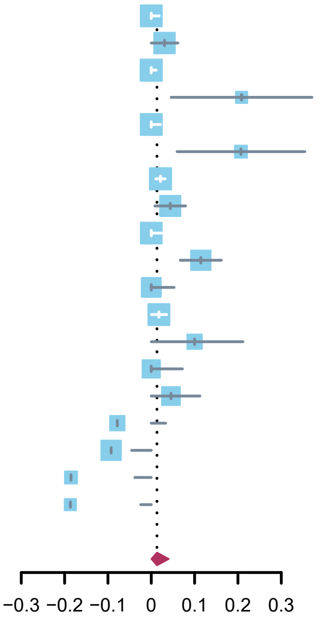

Figure S7. The funnel plot of the pooled results of urinary tract infections after trim-and-fill analysis.

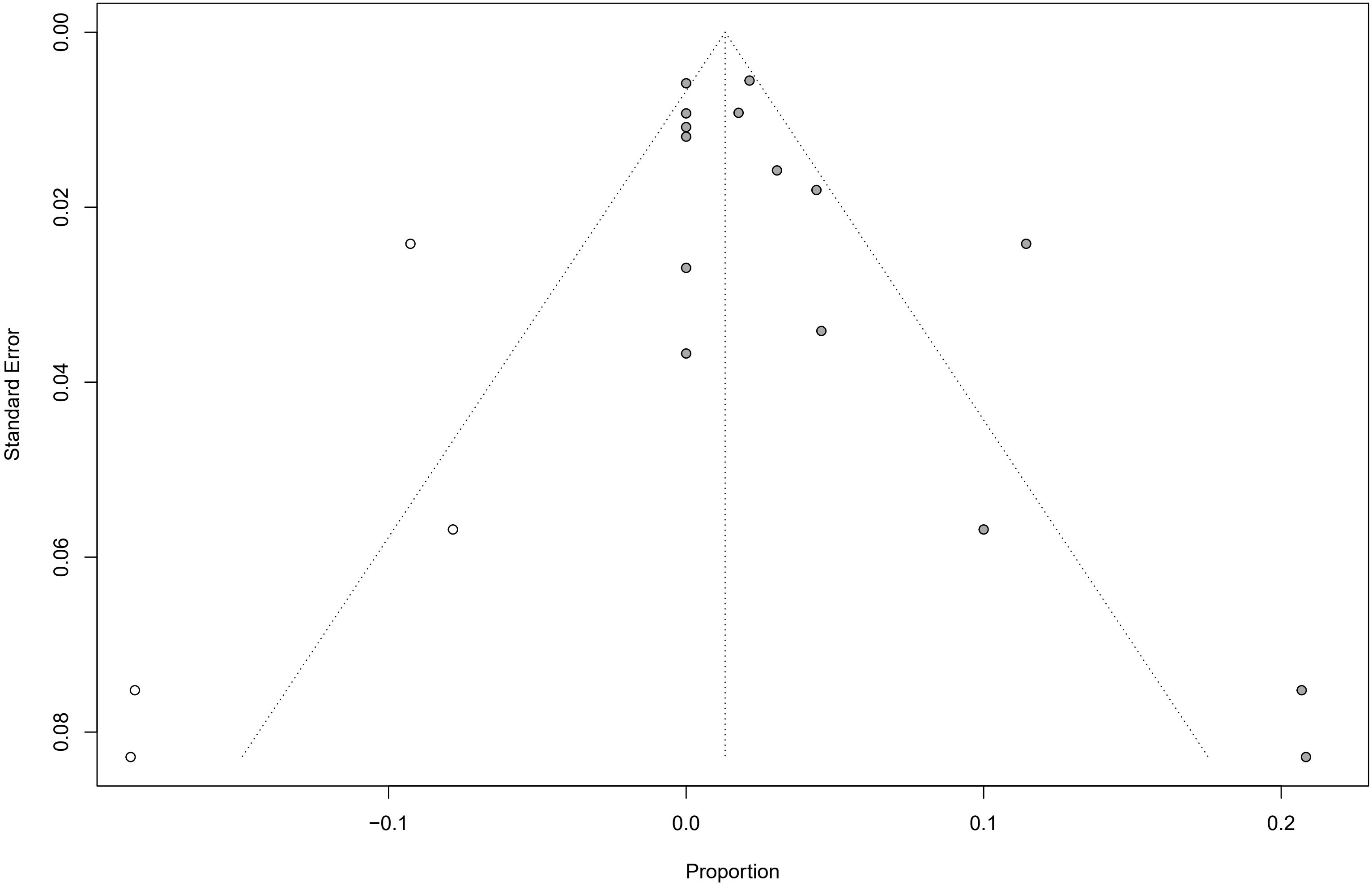

Figure S8. The sensitivity analysis of the pooled results of urethral stricture.

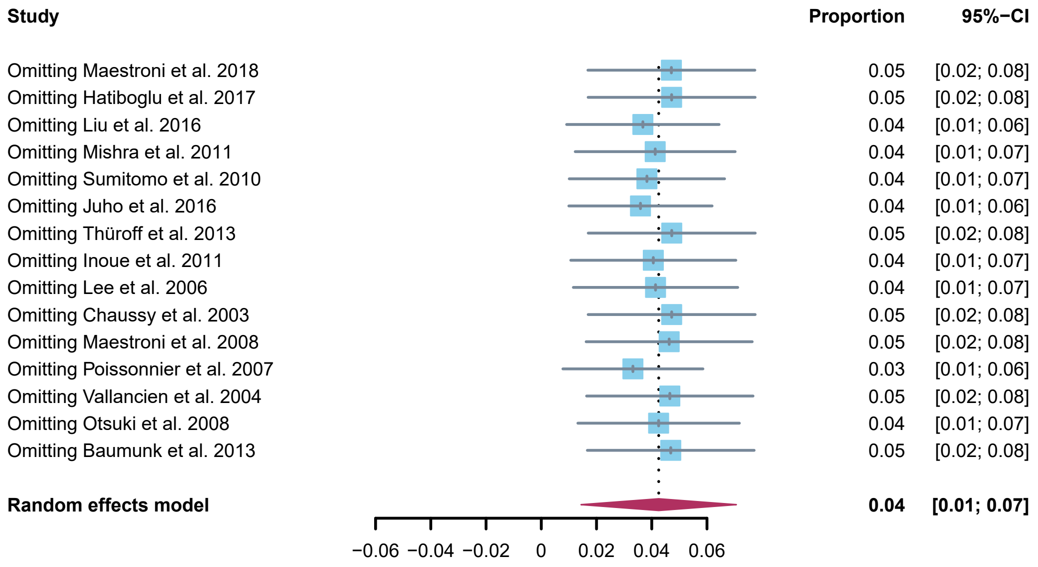

Figure S9. The funnel plot of the pooled results of urethral stricture.

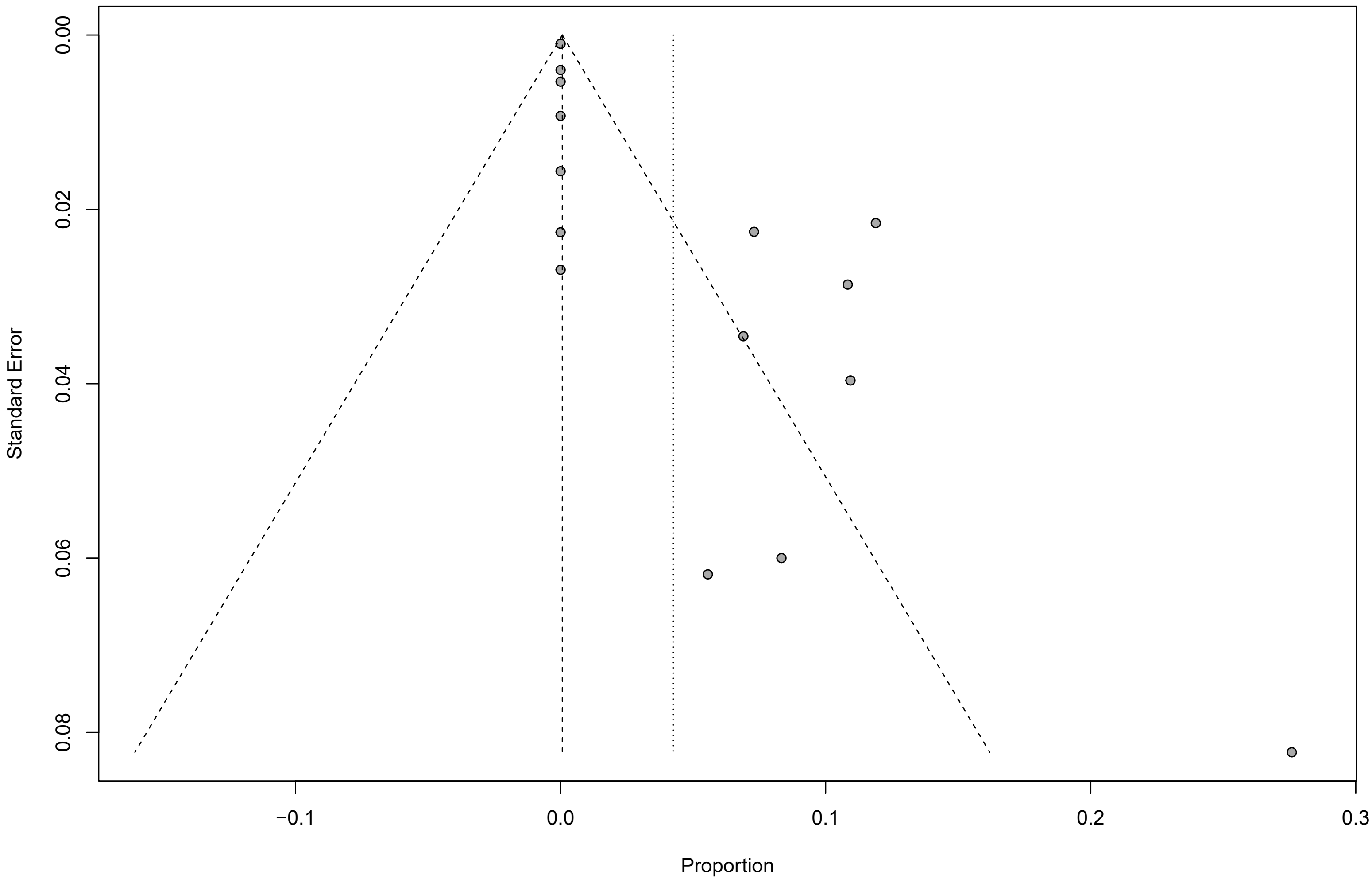

Figure S10. The forest plot of the pooled results of urethral stricture after trim-and-fill analysis.

Study	TE	seTE	Proportion	95%−CI	Weight
Maestroni et al. 2018	0.000	0.0093	0.000	[0.000; 0.018]	6.1%
Hatiboglu et al. 2017	0.000	0.0053	0.000	[0.000; 0.010]	6.1%
Liu et al. 2016	0.108	0.0286	0.108	[0.052; 0.164]	5.5%
Mishra et al. 2011	0.083	0.0600	0.083	[0.000; 0.201]	4.1%
Sumitomo et al. 2010	0.109	0.0396	0.109	[0.032; 0.187]	5.1%
Juho et al. 2016	0.276	0.0823	0.276	[0.115; 0.437]	3.2%
Thüroff et al. 2013	0.000	0.0010	0.000	[0.000; 0.002]	6.2%
Inoue et al. 2011	0.073	0.0226	0.073	[0.029; 0.117]	5.7%
Lee et al. 2006	0.069	0.0346	0.069	[0.001; 0.137]	5.3%
Chaussy et al. 2003	0.000	0.0040	0.000	[0.000; 0.008]	6.1%
Maestroni et al. 2008	0.000	0.0269	0.000	[0.000; 0.053]	5.6%
Poissonnier et al. 2007	0.119	0.0216	0.119	[0.077; 0.161]	5.8%
Vallancien et al. 2004	0.000	0.0226	0.000	[0.000; 0.044]	5.7%
Otsuki et al. 2008	0.056	0.0619	0.056	[0.000; 0.177]	4.0%
Baumunk et al. 2013	0.000	0.0156	0.000	[0.000; 0.031]	6.0%
Filled: Liu et al. 2016	−0.108	0.0286	−0.108	[0.000; −0.052]	5.5%
Filled: Sumitomo et al. 2010	−0.109	0.0396	−0.109	[0.000; −0.031]	5.1%
Filled: Poissonnier et al. 2007	−0.119	0.0216	−0.119	[0.000; −0.076]	5.8%
Filled: Juho et al. 2016	−0.275	0.0823	−0.275	[0.000; −0.114]	3.2%
**Random effects model**			**0.014**	**[0.000; 0.055]**	**100.0%**

Heterogeneity: $I^2 = 87\%$, $p < 0.01$

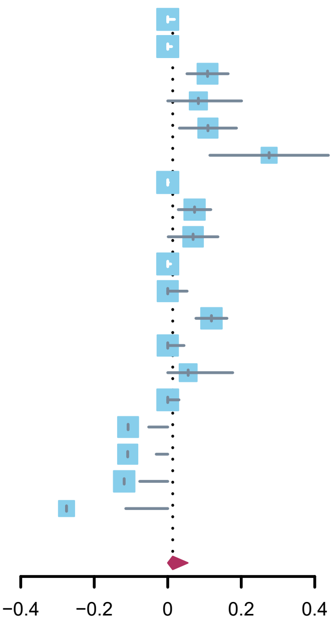

Supplement: Supplementary file 1 [file DataSheet_1.pdf]
